# The Relationship between Personality Traits, the 5HTT Polymorphisms, and the Occurrence of Anxiety and Depressive Symptoms in Elite Athletes

**DOI:** 10.1371/journal.pone.0156601

**Published:** 2016-06-03

**Authors:** Annamaria Petito, Mario Altamura, Salvatore Iuso, Flavia A. Padalino, Francesco Sessa, Giovanna D'Andrea, Maurizio Margaglione, Antonello Bellomo

**Affiliations:** 1 Department of Clinical and Experimental Medicine, Psychiatry Unit, University of Foggia, Foggia, Italy; 2 Department of Clinical and Experimental Medicine, Medical Genetics, University of Foggia, Foggia, Italy; University of Birmingham, UNITED KINGDOM

## Abstract

The purpose of this study was to determine the relationship between personality, the serotonin transporter (5HTT) polymorphisms and the occurrence of anxiety and depressive symptoms in elite athletes. 133 healthy participants completed the NEO Five-Factor Inventory (NEO-FFI). The mood states were assessed using the Profile of Mood States (POMS) questionnaire. The athlete’s mental skills were assessed through the Sport Performance Psychological Inventory (IPPS-48). The occurrence of psychiatric and personality disorders was assessed using the Clinical Interview for DSM-IV Disorders. A polymerase chain reaction was employed to identify genotypes at the 5HTTLPR polymorphism. The 5HTTLPR *s/s* genotype was associated with both neuroticism (p< 0.001) and tension/anxiety symptoms according to the POMS (p<0.02), cognitive anxiety and emotional arousal control according to the IPPS-48 (p<0.01). Significant correlations were proved between neuroticism and symptoms of anxiety and depression (p<0.05). Neuroticism mediates the association between the 5HTTLPR polymorphism and symptoms of cognitive anxiety and emotional arousal control (p<0.05). These results suggest a significant interaction between the 5HTTLPR polymorphism, neuroticism and sport related stress that predict adverse mental health outcomes in athletes. Identification of homogeneous groups of athletes having predispositions to anxiety and depressive symptoms may help to implement early prevention programs.

## Introduction

Many studies investigating the relationship between sport participation and mental health have suggested that participation in sport is associated with psychological benefits including higher levels of self-esteem and lower levels of depression [1, 2]. However, recently, a significant body of research has found evidence of mental health problems associated with sport participation as a result of sport-related significant stressors [3, 4]. A wide range of psychiatric disorders has been reported in the athletic population including depression, anxiety, disordered eating, bipolar disorders and substance abuse [5, 6]. In recent years, the prevalence of depression and anxiety disorders has been reported as increasing in this population [7, 8, 9]. However, the mechanisms leading to both depression and anxiety disorders among athletes are mostly unknown.

Many risk factors have been proposed, such as competition-related stress [10]; ineffective coping strategies [11, 12] overtraining [13, 14]; negative group dynamics/peer interaction [15,16, 17] and negative interactions with leaders [18,19]. Besides the above-mentioned factors, individual aspects should be considered. In this regard, specific personality traits that might have an influence on the development of depressive and anxiety symptoms are of interest.

Personality is a multidimensional structure that is created by both environmental and genetic factors. According to twin and family studies, individual variation of the heritable component is estimated to account for 30–60% of variance in personality traits [20, 21]. Serotonin (5-HT) neurotransmission has a key role in the regulation of the activity of the central nervous and influences a wide variety of physiological and psychological processes including individual differences in personality traits [22, 23].

The serotonin transporter gene (SLC6A4) encodes the serotonin transporter protein (5-HTT), which acts as a key regulator by removing serotonin from the synaptic cleft. The promoter region of the SLC6A4 gene contains a polymorphism with short (s) and long (l) repeats in a region: 5-HTT-linked polymorphic region (5-HTTLPR). The long form was shown to be associated with higher and the short form with lower expression of the gene product [24]. Several studies have provided strong evidence for an association between the 5-HTTLPR short allele and neuroticism, defined as proneness to negative emotionality, including depression and anxiety. Individual with either one or two copies of the short allele had significantly greater levels of neuroticism than those homozygous for the long allele [25].

The analysis of the association between the 5-HTTLPR polymorphism and neuropsychiatric disorders, including anxiety and depressive syndromes, has shown some positive but contradictory findings. Significant associations between the short variant and susceptibility for mood disorders have been reported [26, 27, 28] but other studies did not confirm these findings [29]. The original results of Caspi et al. [30] suggested that individuals carrying the short (*s*) allele are more likely to develop major depression following exposure to early life stress (e.g., childhood maltreatment). More recently, many investigators found that this polymorphism confers risk for psychopathology in the presence of stress during adulthood [31, 32], which has been confirmed in mouse models [33]. Individuals with one or two copies of the *s* allele exhibited higher levels of depressive symptoms following exposure to stressful events than did individuals who were homozygous for the *l* allele. Perhaps most notably, a number of studies have shown that neuroticism mediates the association between the serotonin transporter gene polymorphism and stress-reactive phenotypes like depression and anxiety by increasing the magnitude of emotional reactivity to stressful events [34, 35]. Taken together, these findings suggest that this genetic polymorphism produces an increased sensitivity to the impact of stressful events that in turn increases the likelihood of anxiety and depression.

Although there is evidence that athletes are highly vulnerable for developing mental health problems due to the level of stress they experience [36] little is known about the association between the 5-HTTLPR polymorphism and the development of depressive and anxiety symptoms in the athlete population. Particularly, several studies addressing the topic of depressive symptomatology in elite athletes reported high levels of depressive symptoms, as assessed by self-report measures, rather than major depression diagnoses [37, 38, 39]. These findings suggest that subclinical depression and anxiety (i.e., symptoms not meeting the threshold for a formal DSM diagnosis) should be not neglected in the elite athlete population. There is evidence for an association between anxiety-related personality traits such as neuroticism and depressive and anxiety disorders [40]. In addition research suggests that an episode of major depression increases trait neuroticism and it is possible that the mediating effect of neuroticism on the association between the 5-HTTPRL genotype and depression may be due, in part at least, to the fact that those who report a history of lifetime major depression will also report elevated trait neuroticism, because of this history [41]. Since we know little about this bi-directional causality we decided to reduce the heterogeneity of our sample by including only participants without a history of depressive or anxiety symptoms meeting the threshold for a formal DSM diagnosis. Most studies investigating the influence of serotonin transporter gene polymorphisms and stressful events on affective disorders have used the diagnoses of depressive and anxiety disorders as outcome variables. However, several researchers suggested that depression and anxiety may best be viewed as dimensional, not a categorical, constructs and explored the influence of the 5-HTTLPR genotype on affective disorders treating anxiety and depression as continuous variables [42, 43]. Therefore, in the current study we used measures of depressive and anxiety symptoms as continuous variables as the outcome variables disorders.

The purpose of this study is to determine the relationship between personality, the presence of the polymorphism in the 5HTT (SLC 6A4) promoter region and the severity of anxiety and depressive symptoms in a homogenous group of elite athletes. We hypothesized that the 5-HTT *s* allele as well as neuroticism might influence the development of depression and anxiety disorders in elite athletes engaged in competitive sports. Furthermore, given existing evidence that neuroticism mediates the putative association between 5-HTTLPR genotype and lifetime major depression [25], it is plausible that the personality trait of neuroticism might mediate the association between this gene and symptoms of anxiety and depression. A mediation analysis was used to test this hypothesis.

## Methods

### Subjects

In this study one hundred and thirty-three elite athletes were recruited from national sporting centers in Apulia Region (Italy) between January 2013 and December 2014. An initial letter of invitation to participate in the research project was sent to head coaches as well as to directors of elite sport organizations. 18 centers agreed to participate in the study. Then, we scheduled a meeting with athletes in the sport centers to describe in more details the purpose and the planning of the project. 73% (133/182) of the men elite players and 63% of the female (12/19) chose to participate, as women represent less than 10% of our sample, it was decided to exclude them as it would be very difficult to obtain an adequate sample size. We scheduled evaluations to coincide with the start of players’ season. Participants were healthy men who competed, within the last 5 year, at a national or international level in their chosen sport (soccer, basket ball, hockey), certificated by CONI (Italian National Olympic Committee). All participants were competing in championship competitions when the study was conducted. All participants were informed in details about the purpose of the study. Next, they gave their written consent for taking part in the study, for collecting their blood, as well as storing and subjecting it to a genetic analysis. Exclusion criteria for all participants included a history of traumatic brain injury, epilepsy, developmental disorder, diagnosable current substance abuse dependence or other known neurological condition and the presence or previous presence of psychiatric disorders. The present study is in accordance with the Helsinki declaration and was approved by the local Institutional Review Board (Comitato Etico ASL-FG; prot. n. 09/CE/07).

### DNA analysis

A blood sample was collected in ethylenediaminetetraacetic acid or sodium citrate from each participant and DNA was extracted from peripheral blood leukocytes according to standard protocols [44]. DNA amplification was amplified using the 2 flanking primers suggested in 1996 by Heils et coll: 5-HTTU:5'GGCGTTGCCGCTCTUAATGC3', nt-1416,-1397 5-HTTL:5'GAGGGACTGAGCTGGACAACCAC, nt-910,-889. This set of primers amplifies a 484/528 fragment corresponding to the SLC6A4*C short and long allele, respectively. The PCR conditions were slightly modified from Heils et coll. [45]. The PCR reaction was carried out in a total volume of 20 μL consisting of 100 ng of genomic DNA, 0.1 μmol of primers per liter, 40-μmol/L deoxynucleotide triphosphates, 20-μmol/L 7-deaza-2'deoxyguanosine, and 1 unit of AmpliTaq with the appropriate buffer in a Mastercycler polymerase chain reaction thermal cycler (Eppendorf, Hamburg, Germany). Cycling conditions were as follows: 1 denaturing cycle at 95°C for 5 minutes, 2 cycles with a touchdown annealing temperature of 63°C and 62°C, respectively for 30 seconds, and 38 cycles with an annealing temperature at 61°C. Final DNA elongation was at 72°C for 10 minutes. DNA bands were visualized in prestained (0.4-μg/mL ethidium bromide) 3% agarose gels that were run for 1 hour at 120 V.

### Psychometric Evaluation

The Structured Clinical Interview for DSM-IV Axis I Disorders (SCID-I) [46] and Structured Clinical Interview for DSM-IV Axis II Personality Disorders (SCID II) [47], were administrated to assess current and previous psychiatric diagnoses. The clinical assessment was conducted by psychiatrists and/or licensed research psychologists who were trained to a minimum interclass correlation of 0.80. The NEO Five-Factor Inventory (NEO-FFI) was used to assess of personality [48]. The personality description is given in 5 dimensions: neuroticism, extraversion, agreeableness, openness and conscientiousness. The structure of this test has been validated in a variety of populations and cultures [49, 50, 51] using various personality inventories [52]. The Sport Performance Psychological Inventory (IPPS-48) and the Profile of Mood States (POMS) self-reporting questionnaires were used to assess anxiety and depressive symptoms. The IPPS-48 includes 48 items pertaining to eight factors. These factors are further included into two broader conceptual categories. The Cognitive aspects category encompasses race preparation (RP), goal-setting (GS), mental practice (MP), and self-talk (ST) factors. The Emotional aspects category comprises self-confidence (SC), emotional arousal control (EAC), cognitive anxiety (CA) or worry and concentration disruption (CD). The IPPS-48 possesses good psychometric properties (alpha and test-retest reliability), and discriminant validity [53]. The POMS is a standard validated psychological test formulated by McNair et al. [54] consisting of 65 items that fit into 6 categories: tension-anxiety (T/A), depression-dejection (D/D), anger-hostility (A/H), vigor-activity (V/A), confusion-bewilderment (C/B) and fatigue-inertia (F/I). The Profile of Mood States (POMS), developed as a means of measuring the current mood of patients, has often been employed in studies measuring athletes’ emotional states [55]. Interviews and testing were arranged at times convenient for the participants. One hour prior a competition, all participants were also interviewed about the feeling of stress in their own terms [56]. These unstructured interviews began with an open-ended prompt designed to guide the direction of the interview: “Tell me about your experience of stress as an athlete”.

### Statistical Analysis

Analyses were conducted using the statistical software Grand Prism 5 (San Diego, CA, USA). Means and SD have been calculated for each studied parameter, and an alpha level of 0.05 was selected throughout the study. The differences in psychometric dimensions across the three 5HTT-LPR genotype were compared using nonparametric Kruskal Wallis test with Dunn's multiple Comparison post-hoc testing. The assessments of the relationship between dimensions of temperament (NEO-FFI) and mood states (POMS and IPPS-48) were performed using Pearson’s correlation. Conformity of empirical genotype frequency distribution to theoretically expected Hardy–Weinberg equilibrium was verified using Pearson χ2 test.

### Mediation analyses

**Fig 1:**
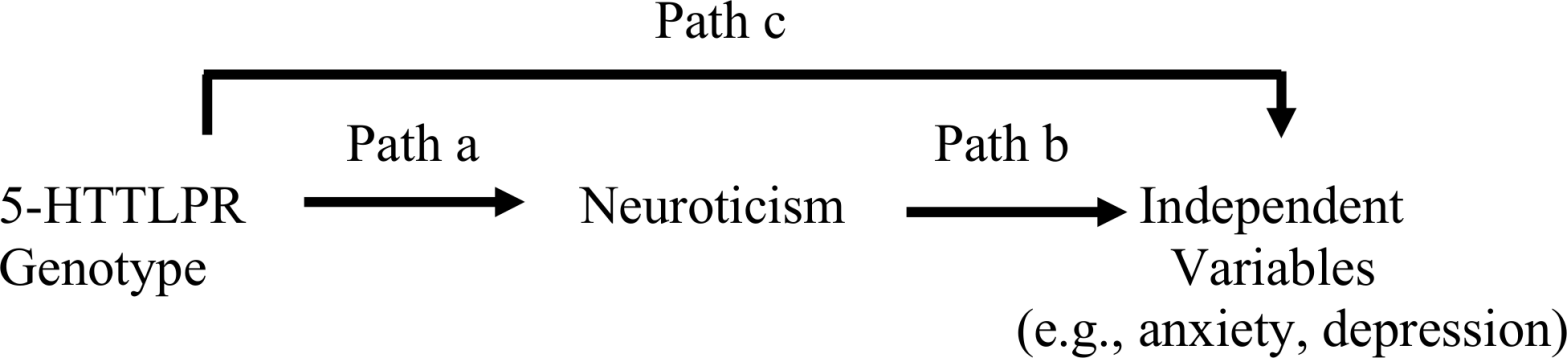
Paths in mediation model.

A path method was applied to test the hypothesis that the association between 5-HTTLPR genotype and symptoms of anxiety and depression was mediated by trait neuroticism. In particular a series of linear regression models was run to assess the fourth criterion for mediation proposed by Baron and Kenny [57] as described by Frazier et al. [58]. This approach involves testing three equations. First, the outcome variable is regressed on the predictor (Path c) (Fig 1). Second, the mediator is regressed on the predictor variable (Path a). In the third equation, the outcome variable is regressed on both the predictor and the mediator. This provides a test of whether the mediator is related to the outcome (Path b) as well as an estimate of the relation between the predictor and the outcome controlling for the mediator (Path c'). If the relation between the predictor and the outcome is significantly smaller when the mediator is in the equation (Path c') than when the mediator is not in the equation (Path c), the data suggest a mediation effect [58]. Therefore, first, the IPPS-48, POMS scores (the outcome variables) were regressed on the 5-HTTLPR genotype (the predictor; coded as 0 for ‘ll’, 1 for ‘ls’, and 2 for ‘ss’) to establish that there was an effect to mediate (Path c). Second, the neuroticism (NEO-FFI) score (the mediator) was regressed on the 5-HTTLPR genotype (the predictor variable) to show that the predictor was related to the mediator (Path a). Finally, the IPPS-48 and POMS scores (the outcome variables) were regressed on both the 5-HTTLPR genotype (the predictor) (Path b) and the neuroticism (NEO-FFI) score (the mediator) (Path c'). To assess the significance of the mediating variable effect we used the method proposed by Baron and Kenny [57]. Specifically, the product of paths a and b is divided by a standard error term. The mediated effect divided by its standard error yields a *z* score of the mediated effect. If the *z* score is greater than 1.96, the effect is significant at the .05 level.

## Results

Participants had no history of neurologic, psychiatric disorders or alcohol and other drug-dependence disorders. The average age of the participants was 23.36 (SD = 8.48). ranged from 18–36 years. Mean educational level was 14.41 (SD = 2.42). The genotype subgroups did not differ significantly in age (s/s = 24.52 (SD = 4.56); s/l = 22.79 (SD = 3.86); l/l = 24.06 SD = 4.24) and education years (s/s = 14.75 (SD = 2.36); s/l = 14.05 (SD = 2.54); l/l = 14.44 (SD = 2.36) (all p > 0.05). All participants reported high levels of stress related to competition (e.g. pressure to perform well and pressure of meeting expectations). 5-HTTLPR genotype and allele frequencies are shown in Table 1.

**Table 1:** Frequencies of the genotypes and alleles of the 5-HTTLPR.

Genotypes			Alleles	
*l/l*	*l/s*	*s/s*	*l*	*s*
(N = 37)	(N = 74)	(N = 22)		
0.28%	0.56%	0.16%	0.56%	0.44%

The distribution of allele in the sample was in the Hardy-Weinberg equilibrium (p > 0.05). The mean scores on the five personality sub-scales of the NEO-FFI for each genotype group are presented in Table 2.

**Table 2:** Association between 5-HTTLPR genotype and *NEO Five-Factor Inventory* (NEO-FFI). Mean ± SD. P = level of significance.

Genotype	N	Neuroticism	Extraversion	Openness	Agreeableness	Coscientiousness
*l/l*	37	14.40± 6.69	32.27 ± 4.22	24.77 ± 4.68	27.50 ± 3.79	32.81 ± 5.99
*l/s*	74	17.47 ± 8.93	31.62 ± 4.71	24.57 ± 5.72	28.88 ± 10.87	34.47 ± 7.57
*s/s*	22	22.49 ± 6.55	29.11 ± 4.22	25.26 ± 4.59	26.68 ± 4.09	32.32 ± 6.78
group S (*l/s* + *s/s*)	96	18.62 ± 8.68	31.04 ± 4.70	24.73 ± 5.47	28.37 ± 9.76	33.98 ± 7.42
P		0.001	0.052	0.689	0.791	0.671

The analyses across the 5HTT-LPR genotype groups (group S (s/s + s/l); l/l; l/s; s/s) indicated a significant main effect of the *s/s* genotype on neuroticism (χ² (3) = 16.03, p = 0.001). Post-hoc analyses revealed a recessive effect of the short allele of the 5-HTTLPR gene with increased neuroticism score in the s/s genotype group and in the S group compared with the s/l and l/l groups (all, p < 0.05). There was no association with the other major personality sub-scales of the NEO-FFI. The mean scores on sub-scales of the POMS and IPPS for each genotype group are presented in Tables 3 and 4.

**Table 3:** Association between 5-HTTLPR genotype and Profile of Mood States (POMS). Mean ± SD. P = level of significance.

Genotype	N	Tension-Anxiety	Depression-Dejection	Anger-Hostility	Confusion-Bewilderment	Fatigue-Inertia	Vigor-Activity	Negative-Mood-States	Total
*l/l*	37	5.00±3.37	4.56±4.78	6.60±6.68	5.84±4.03	6.88±5.24	19.68±6.32	28.88±14.58	9.22±16.22
*l/s*	74	7.37±5.38	4.98±6.22	6.58±6.21	5.88±4.37	8.02±6.25	17.63±6.93	32.83±20	15.2±23.16
*s/s*	22	9.56±5.48	9.89±10.95	11.61±11.38	7.33±6.45	8.11±5.62	18.28±3.74	46.5±33.67	28.22±34.04
group S(*l/s* +*s/s*)	96	7.78± 5.45	6.11±7.77	7.74±7.92	6.22±4.92	8.04±6.08	17.78±6.33	35.98±24.29	18.2±26.41
P		0.028	0.674	0.665	0.987	0.842	0.526	0.126	0.084

**Table 4:** Association between 5-HTTLPR genotype and Sport Performance Psychological Inventory (IPPS-48). Mean ± SD. P = level of significance.

Genotype	N	RP	ST	CA	SC	GS	EAC	MP	CD	Cognitive-Aspects	Emotional -Aspects
*l/l*	37	25.27±7.77	20.23±8.36	15.0±5.83	27.88±8.9	22.46±6.18	24.69±4.44	21.9± 7.92	11.69±4.58	89.92±1.45	25.88±14.7
*l/s*	74	24.37±6.19	22.37±7.88	18.59±7.40	24.49±8.47	22.76±8.25	22.05± 6.07	20.69± 8.01	11.05±5.34	90.19 ±19.28	17.31± 19.11
*s/s*	22	25.0±74.41	25.0±7.34	21.89±7.38	25.61±7.59	17.89±7.39	21.56± 2.08	20.22± 8.50	12.89± 3.22	88.11± 13.9	12.39± 11.75
group S (*l/s* +*s/s*)	96	24.51±5.82	22.96±7.8	19.32±7.48	24.74±8.46	21.62±8.27	21.94± 5.4	20.58±8.07	11.48±4.97	89.67± 18.16	15.88±17.92
P		0.2031	0.3258	0.0128	0.9951	0.3258	0.0392	0.6134	0.0977	0.8551	0.0025

RP = Race Preparation; ST = Self-Talk; CA = Cognitive Anxiety; SC = Self-Confidence; GS = Goal-Setting; EAC = Emotional Arousal Control; MP = Mental Practice; CD = Concentration Disruption

There was a significant main effect of 5-HTTLPR genotype on anxiety symptoms according to the POMS (χ² (3) = 9.034, p = 0.02). Post-hoc analyses revealed that participants with two copies of *s* allele had significantly higher POMS tension /anxiety (T/A) scores than those with the l/l genotype (p<0.05). Furthermore, we found a main effect of 5-HTTLPR polymorphism on cognitive anxiety (χ² (3) = 10.81, p = 0.01) and emotional arousal control (χ² (3) = 8.358, p = 0.03) according the IPPS-48. Post-hoc analyses revealed that participants with two copies of *s* genotype had higher IPPS cognitive anxiety and lower IPPS emotional arousal control scores than those with the l/l genotype (all, p<0.05). A significant correlation was observed between anxiety and depressive symptoms according to the POMS (tension/anxiety, depression/dejection, anger/hostility) and IPSS (cognitive anxiety, emotional arousal control, concentration disruption) and neuroticism according to the NEO-FFI (Table 5).

**Table 5:** Correlation (Pearson’s r) between and Sport Performance Psychological Inventory (IPPS), Profile of Mood States (POMS) and Neuroticism.

	Neuroticism	
	Correlation-coefficient	P value
POMS Tension-Anxiety	0.36	0.0001
POMS Depression-Dejection	0.23	0.0175
POMS Anger-Hostility	0.27	0.0063
POMS Vigor-Activity	-0.08	0.4183
POMS Fatigue-Inertia	0.19	0.0564
POMS Confusion-Bewilderment	-0.03	0.7793
IPPS Race Preparation	-0.13	0.1967
IPPS Self-Talk	0.07	0.5080
IPPS Cognitive Anxiety	0.21	0.0368
IPPS Self-Confidence	0.15	0.1165
IPPS Goal-Setting	-0.13	0.1818
IPPS Emotional Arousal Control	-0.24	0.0112
IPPS Mental Practice	-0.10	0.1486
IPPS Concentration Disruption	0.21	0.0320
IPPS Cognitive Aspects	-0.08	0.4219
IPPS Emotional Aspects	-0.01	0.9928

The results of regression analyses are summarized in Table 6. These analyses indicated that the criteria proposed for mediation by Baron and Kenny [57] were met for a mediating influence of neuroticism on the association between 5-HTTLPR genotype and tension/anxiety, as measured with POMS, and cognitive anxiety and emotional arousal control as measured with IPPS. To establish the statistical significance of the difference between paths c and c’ we used the Baron and Kenny’s test [57] as described earlier. The application of this test yielded the following results: the drop of the unstandardized regression coefficient associated with cognitive anxiety in the models without (B = 3.51) and with neuroticism (B = 0.31) (i.e., from c to c') was significant (p < .05). The drop of the unstandardized regression coefficient associated with emotional arousal control in the models without (B = -1.74) and with neuroticism (B = -0.16) also was significant (p < .05); while the drop of the unstandardized regression coefficient associated with tension/anxiety in the models without (B = 0.135) and with neuroticism (B = 0.08) was not significant (p>.05).

**Table 6:** Mediator Effects Using Multiple Regression.

Path c	B	*SE* B	95%CL	β	P
Outcome: Tension-Anxiety					
Predictor: 5HTT genotype	0.13	0.06	1.45,1.74	0.17	0.03
Outcome: Cognitive-Anxiety					
Predictor: 5HTT genotype	3.51	0.86	13.0,16.8	0.33	0.0008
Outcome: Arousal-Control					
Predictor: 5HTT genotype	-1.74	0.64	22.6,25.4	-0.23	0.007
**Path a**					
Outcome: Neuroticism					
Predictor: 5HTT genotype	3.60	1.04	12.3,16.9	0.28	0.0007
**Paths b and c'**					
Outcome: Tension-Anxiety					
Mediator: Neuroticism (Path b)	0.01	0.005	1.50,1.91	0.22	0 .001
Predictor: 5HTT genotype	0.08	0.06	1.41,1.73	0.11	0.02
Outcome: Cognitive-Anxiety					
Mediator: Neuroticism (Path b)	2.39	0.06	7.71,13.0	0.36	0.00001
Predictor: 5HTT genotype	0.31	0.84	13.1,16.8	0.11	0.08
Outcome: Arousal-Control					
Mediator: Neuroticism (Path b)	-1.13	0.64	24.5,28.5	-0.15	0.00001
Predictor: 5HTT genotype	-0.16	0.06	22.6,26.0	-0.28	0.005

Only the outcome variables entering the final model are shown. B = unstandardized regression coefficient; *SE* = Standard Error; CL: Confidence Limits; β = standardized regression coefficient.

## Discussion

We found evidence for an association between a serotonin transporter promoter polymorphism (5-HTTLPR), personality trait of neuroticism, and symptoms of depression and anxiety among elite athletes. Particularly, individuals with 5-HTTLPR s genotypes (s/s, s/l) had increased scores for anxiety as measured with POMS, increased scores for cognitive anxiety and decreased scores for emotional arousal control as assessed with IPPS. In addition, individuals with 5-HTTLPR s genotypes had greater level of neuroticism, as measured by the NEO-FFI, than their counterparts with two copies of *l* allele. A significant correlation was observed between neuroticism and the severity of negative emotionality according to the POMS (tension-anxiety, depression-dejection, anger-hostility) and the IPPS (cognitive anxiety or worry, emotional arousal control, concentration disruption).

The association between the 5-HTTLPR s*s* genotype with anxiety symptoms and decreased emotional arousal control is not unexpected in view of the results of previous studies that revealed a small but significant relationship between the 5-HTTLPR polymorphism and anxiety and depression disorders [25, 59, 60, 61, 62]. Recently, a number of studies suggested that biological stress reactivity may be a critical mechanism underlying the association between the serotonin transporter gene and exposure to stressful events in increasing risk for depression [31, 32]. Consistent with this “stress reactivity” several investigators found that, compared with individuals who have an *l* allele, individuals homozygous for the *s* allele exhibit greater amygdala activation in response to fearful stimuli [63, 64] and had higher cortisol levels following exposure to a stressor [31, 65]. Anxiety is associated with raising the level of activation of the body, muscle tension, difficulty and coordinative disturbances in focusing attention, especially when the athlete perceives not have control of the situation [66]. Emotional arousal control contributes managing emotions and channeling energies towards the performance, especially in situations of high stress competitive [67]. Therefore, our findings suggest that athlete with one or two copies of *s* allele were more likely then were their counterpart with two copies of l allele to became anxious in response to stressful competitive experiences.

Several studies suggest that 5-HTTLPR genotype predicts reciprocal associations between environmental stress and depressive symptoms in non-patient populations [41, 68]. Specifically, those studies suggest that high environmental stress interacted with the 5-HTTLPR genotype to predict subclinical depression symptoms such that those with the *s* allele had significantly higher depression ratings, as compared to those with the *l* allele. The current study expands upon a growing literature showing that the *s* allele was associated with symptom of anxiety and depression in athletes who are exposed to tremendous psychological stress on a daily basis. The possible determinants of depression and anxiety symptomatology among elite athletes (e.g. cost and effort of the exercise), and the degree to which they may or may not have been experienced, were unexplored in our study, as the focus of the current study was on the experience of depression itself, not its causes. However, it is worth noting that the association between stress related to competition (e.g., pressure to perform well and pressure of meeting expectations) and symptoms of anxiety and depression in our sample is consistent with the results of previous studies that reported that sport specific demands (such as overwhelming demands of training, psychological pressure or failure) are associated with higher levels of depressive symptoms in elite athlete [37, 69].

The association between neuroticism and 5-HTTLPR genotypes is in line with the results of previous studies that revealed a relationship between the 5-HTTLPR polymorphism and anxiety-related personality traits [25, 70]. Neuroticism may contribute to the occurrence of anxiety and depressive symptoms in elite athletes dealing with competitive situations [40]. According to the results of the current study the influence of genetic variation on anxiety and emotion regulation are mediated by neuroticism. First, 5HTTLPR genotype was significantly associated with both neuroticism and symptoms of anxiety and poor emotional control. Second, neuroticism was also significantly associated with anxiety and poor emotional control. Finally, when the association between neuroticism and symptomatology was controlled for, the strength of association between 5HTTLPR genotype and anxiety and emotional symptoms was significantly reduced. In particular our results suggest that the association between 5HTTLPR and symptoms of cognitive anxiety and poor emotional control may be partially mediated by trait neuroticism in elite athletes. This is consistent with the results of previous studies suggesting that personality trait of neuroticism mediates, at least in part, the association between 5HTTLPR genotype and affective disorders [35, 71, 72]. Interestingly, it has been suggested that cognitive anxiety in response to stressful events may mediate the relation between neuroticism and the development of depressive symptoms [73]. These stress-related vulnerabilities associated with neuroticism might be influenced by the participation of the athlete at the highest competitive levels [74].

This study has a number of limitations. First, the assessment of level of stress is based exclusively on self-report rather than clinical interview. Therefore, it will be important to replicate the findings using additional standardized behavioral measures of psychological stress. Second, it would increase the value of this research if the study group was more numerous and adverse life events (e.g., childhood maltreatment) [30], potentially contributing to the incidence and severity of depressive and anxiety symptoms, were taken into consideration. However, from a statistical point of view we employed statistical tests that do not assume equal variance between groups. Third, the findings presented in this article only concern participants without a clinical diagnosis of depression or anxiety disorder; indeed, none of the participants had experienced any previous Axis I and Axis II disorder. Perhaps, the inclusion of such subjects in the study would reveal significantly stronger relationships between the variables analyzed than it was observed in this study. Forth, participants were recruited to investigate possible associations among genotype, personality, exposure to stress, and probability of depression and anxiety disorders. It is possible that trait neuroticism and depression or anxiety symptoms may have been associated with participation, so that our sample may have represented a selected sub-sample of the original population. This may reduce the generalizability of our results. However, there are no grounds for supposing that the association between 5-HTTLPR genotypes, neuroticism and both depression and anxiety symptoms should operate differently in participants compared with compared with non-participants.

Despite these limitations, the present study provides evidence in regard to the associations between the 5-HTTLPR *s/s* genotype and anxiety, depression symptomatology in elite athletes. Furthermore, we found that neuroticism plays a significant role in the etiology of anxiety and depressive symptoms that might develop in response to stressful sport related events. Despite its all limitations this is an innovative study presenting a new research area and suggesting the direction of further investigation. In conclusion, we have demonstrated a significant relationship between personality, the presence of the 5-HTTLPR polymorphism, and the severity of anxiety and depressive symptoms among elite athletes, an observation that mirrors previous work demonstrating that the same polymorphism predispose to negative psychiatric outcomes in the presence of stressors. This is the first study, to the best of our knowledge, to find a relationship between a gene, anxiety-related personality traits and the severity of negative emotionality among elite athletes.
